# Assessment of polytetrafluoroethylene composites for deep groove ball bearing applications through free run and rolling contact fatigue tests

**DOI:** 10.1038/s41598-025-94547-4

**Published:** 2025-03-24

**Authors:** Dhruv Deshwal, Sachin U. Belgamwar, Siddappa I. Bekinal

**Affiliations:** 1 Department of Mechanical Engineering, Birla Institute of Technology and Science Pilani, Pilani Campus, Pilani, Rajasthan 333031 India; 2 Department of Mechanical and Industrial Engineering, Manipal Institute of Technology, Manipal Academy of Higher Education, Manipal, Karnataka 576104 India

**Keywords:** PTFE, Tribology, Friction, Wear, Composites, Ball bearings, Engineering, Mechanical engineering

## Abstract

Polytetrafluoroethylene (PTFE) is widely used in tribological applications. However, its usage in the ball-bearing application has not been explored. The present study investigates the usage of PTFE composites for deep groove ball (DGB) bearings. Initially, a ball-on-disc experiment is conducted to analyze the interaction between pure PTFE, 40% bronze-filled, 25% glass fiber-filled PTFE composites, and Si_3_N_4_ balls by determining the coefficient of friction (COF) and specific wear rate. Then, the reliability of the fabricated DGB is checked through free run and rolling contact fatigue (RCF) tests using a 6-channel DAQ system (OR35-INST). In the free run test, a rotor supported on fabricated polymer bearing sets is rotated up to two million cycles under minimal load conditions. Its vibrational signals and RPM are continuously monitored through an accelerometer and tachometer. After a subsequent number of cycles, the dynamic characteristics of the shaft are recorded using the DAQ system and eddy probes. Finally, an RCF test is conducted to check the load-bearing capacity of the bearing. The bearing is subjected to a 36 N to 108 N (in step of 36 N) load, and the vibration signals are recorded.

**Supplementary Information:**

The online version contains supplementary material available at 10.1038/s41598-025-94547-4.

## Introduction

Over the past decade, polymers have gained scope in bearing applications. The polymer bearing offers advantages over traditional metal-based bearings, particularly in extreme conditions. Its self-lubrication properties eliminate the need for external lubrication, enhancing its versatility and reliability in challenging environments^1,2^. Nylon, polyether ether ketone (PEEK), polyphenylene sulfide (PPS), and polytetrafluoroethylene (PTFE) are among the primary polymers utilized in bearing applications. PTFE is the most suitable polymer for these applications due to its unique properties^3^. It provides a low weight ratio, is highly resistant to chemicals^4,5^, and environmental degradation^6–8^. It offers a low COF during interaction due to its self-lubricating properties^9^ and has high thermal stability^10^. It operates effectively across a wide temperature range, from − 260 °C to 260 °C, making it a reliable choice for bearing applications^11^. However, a high wear rate is a significant concern with PTFE, which can be addressed adequately by reinforcement of the filler material to its matrix^12–14^. Various reinforcing materials, such as ceramics^15–17^, fibres^18–20^, nanomaterials^21,22^, metals^23,24^ and polymers^25–27^, have been found suitable to enhance its wear resistance. Selecting appropriate filler material to enhance the PTFE performance can be challenging due to the numerous factors influencing composite performance. Key considerations include speed, load, temperature, environmental conditions, lubrication, the nature of the counter body, and surface roughness. The current study uses PTFE, and its composites filled with 40% bronze (B40) and 25% glass fiber (G25) for ball-bearing applications. Glass fiber is commonly used as a reinforcing agent within fibrous reinforcement materials. Its incorporation significantly enhances mechanical properties, improves creep resistance, and reduces counter-surface adhesion, contributing to a lower wear rate and high load-bearing capacity^28,29^. In contrast, among the various metals and their constituent alloys, bronze is extensively used as a reinforcing agent within the PTFE matrix. Bronze particles serve as a protective barrier, stopping significant fragmentation of PTFE. This results in a considerable decrease in the wear rate^30,31^.

The lower COF in PTFE is because of its ability to transfer the film on the counter body during the interaction, which reduces the direct contact of the polymer with the counterpart. However, the reinforcement can increase PTFE COF during the interaction because filler material acts as a barrier, and effective film transfer may not take place^32,33^. Several articles have investigated using PTFE filled with glass fibre and bronze in bearing applications. Duman et al. have shown that employing bronze in conjunction with PTFE produces superior results compared to the Babbitt alloy. Consequently, the PTFE/bronze composite is deemed suitable for bearing pads^34^. Further, a comparative study analysed PTFE’s friction and wear characteristics reinforced with glass fibre and bronze. Incorporating 17% glass fiber into PTFE resulted in the most significant reductions in both wear rate and friction coefficient, outperforming the 25% bronze-filled PTFE. The wear rate values for both PTFE composites ranged from 10^−8^ to 10^−9^ mm^2^/N, while pure PTFE exhibited a wear rate of 10^−7^ mm^2^/N^35^. Ünlü et al.^36^ have tested pure PTFE, 35% graphite filled, 60% bronze filled, and 25% glass fibre filled PTFE composite in a bearing test rig in dry condition. Results show that the glass fiber-filled PTFE composites suffered the lowest wear rate but showed the highest COF during the interaction. The COF obtained for bronze-filled PTFE was almost like PTFE but had a higher wear rate than glass fibre-filled PTFE.

Prior to utilising the material for a particular application, it is essential to perform a preliminary test. Different tribological tests are available to evaluate PTFE composites’ friction and wear performance for a specific application. However, the ball-on-disc tribological test is particularly well-suited for ball-bearing applications. This testing methodology closely resembles the interaction between the ball and bearing races, ensuring a comprehensive performance assessment under relevant conditions. However, the utilisation of PTFE composites hasn’t been explored for the ball bearing application, and in the past, the PEEK-based polymer has been tested^37,38^. Hence, in the present study, in the initial phase of the evaluation, a ball-on-disc test was performed to assess the compatibility of the composite materials by measuring the COF and the wear rate. Talat Tevrüz has suggested that the true performance of the composite can only be achieved when tested in actual applications, as tribological tests alone will not be sufficient^13^. Further, the proposed research work contributes to fabricating B40, G25, and PTFE DGB bearings using Si_3_N_4_ balls as rotating elements. Si_3_N_4_ is known for its high crushing strength, robustness to the bearing, and non-corrosive nature. Subsequently, bearing characterization was conducted through the free run and RCF tests. In the free run test, the rotor was supported by the fabricated bearings and rotated for 2 million cycles under minimal load conditions. The vibration amplitude and RPM were continuously monitored throughout the test using OR35-INST, a 6-channel DAQ system. Additionally, the dynamic characteristics of the rotor were measured at a particular RPM using eddy current probes and a DAQ system. Finally, an RCF test was executed in which the bearing was subjected to a radial load ranging from 36 N to 108 N throughout 1 million rotations, during which the amplitude and RPM of the rotor were meticulously recorded. The selection of speed and load is as per polymer-based ball bearings available in the market space^39,40^.

## Experimental procedure

### Materials

This study evaluates the wear performance of Pure PTFE, B40, and G25 composites. The associated material properties are summarized in Table 1. The composite rods were procured from Hindustan Nylons, Miraj, India. A rod with a diameter of 40 mm and a length of 200 mm was produced using a cold compression moulding process at a pressure of 300 kg/cm^2^, followed by a sintering process at 370 °C. A Si_3_N_4_ ball used in the test was procured from N. Gandhi & Co. in Mumbai, India, and its properties are also detailed in Table 1.

**Table 1:** Material properties.

Properties	Pure PTFE	PTFE + Bronze (40%)	PTFE + Glass Fiber (25%)	Silicon Nitride Ball
Designation	PTFE	B40	G25	Si_3_N_4_
Density (g/cm^3^)	2.14	2.96	2.23	3.20
Tensile Strength (MPa)	21.08	16.67	13.53	700
Elongation	260%	276%	162%	–
Young’s Modulus (GPa)	0.4	1.37	1.65	310
Poisson’s Ratio	0.46	0.46	0.46	0.29
Hardness	54 Shore D	63 Shore D	65 Shore D	2477 HV
Service Temperature	260	250	250	1100

### Ball-on-disc test

A ball-on-disc test was conducted using the Ducom TR-20 NEO Series friction and wear monitor. A schematic view of the machine is shown in Fig. S1. The PTFE composite disc was prepared from the procured rod with the help of a multi-axis computer numerical control (CNC) machine. The disc measures 40 mm in diameter and 8 mm in thickness. Before the test, the disc surface was smoothed with a surface polishing machine. The PTFE composite discs were subjected to loads ranging from 25 N to 75 N in increments of 25 N. The contact pressure at the point of contact is detailed in Table 2. A Si_3_N_4_ ball of diameter 6.35 mm was used, and the discs were rotated at a speed of 800 RPM with a wear track diameter of 20 mm under dry conditions. The surface roughness of the disc was measured with the help of a Mitutoyo surface roughness tester. PTFE, G25, and B40 discs have an average roughness value of 0.77 μm, 1.055 μm and 0.5985 μm, respectively. The test was conducted at 24 °C and a relative humidity of 35%. The disc’s morphology was also analyzed by FEI-Apreo-S field emission scanning electron microscope (FESEM). The specific wear rate was calculated based on the mass loss observed during the tests, as outlined in Eq. (1).

1 $$WR=\frac{\varDelta\, V}{F \cdot D}$$

Where, *ΔV* = Volume loss (g/mm^3^), *F* = Normal Load (N), *D* = Sliding distance (m).

**Table 2:** Contact pressure at different loading conditions.

Load (*N*)	Contact pressure (MPa)		
	Pure PTFE	PTFE + Bronze (40%)	PTFE + Glass Fiber (25%)
25	50	113	128
50	63	142	161
75	72	163	184

### Bearing fabrication

The bearing parts (inner ring, outer ring, and cage) were fabricated from procured composite rods through a multi-axis CNC machine. Each assembled bearing incorporated 11 silicon nitride balls of 5/32″ diameter. The dimensions of the fabricated bearings match those of the SKF-61804 DGB bearing, featuring an outer diameter of 32 mm, an inner diameter of 20 mm, and a width of 7 mm. The views of the assembled fabricated bearings are shown in Fig. S2.

### Free run test

**Fig. 1:**
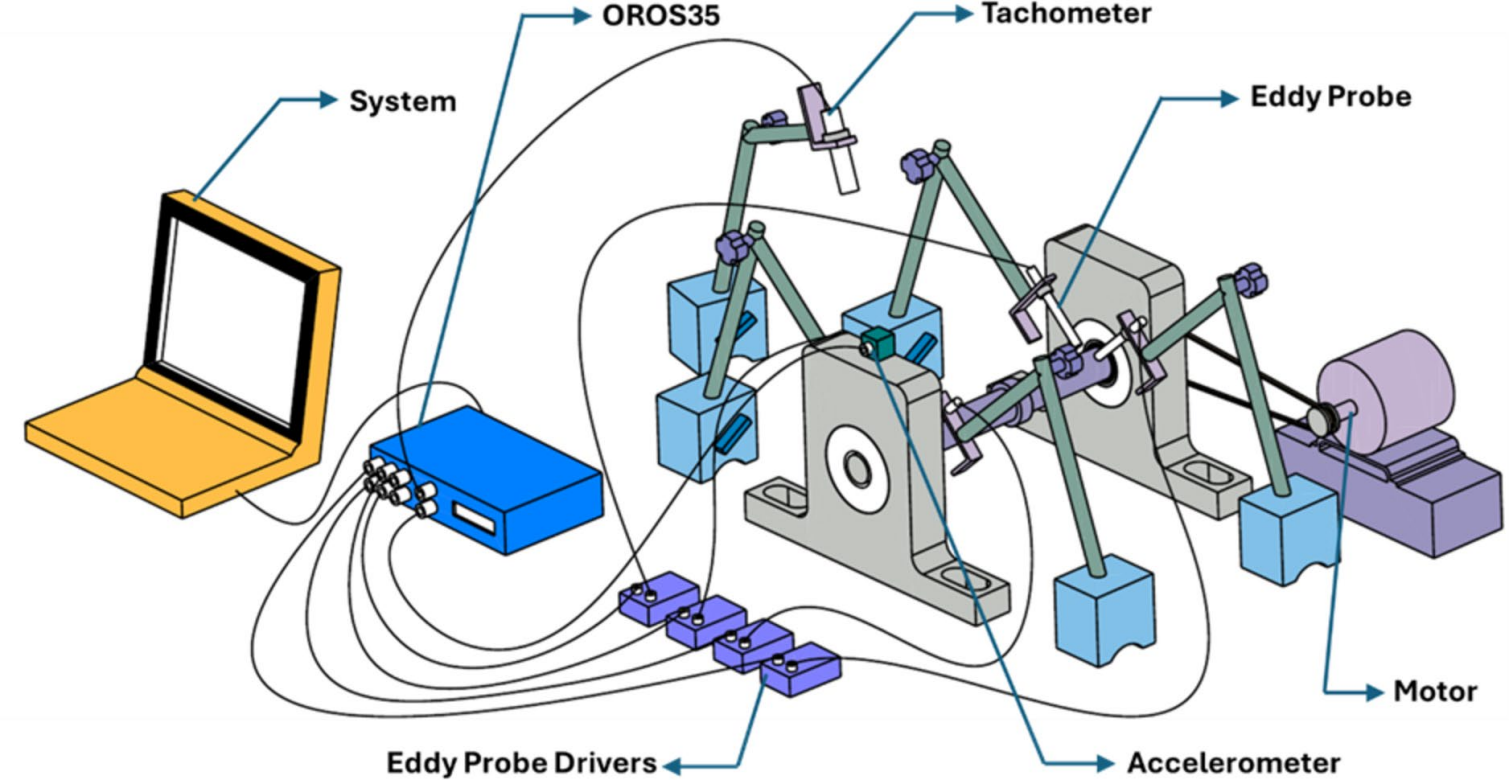
Detailed schematic setup of the free run test (Generated through CATIA 3Dexperience https://www.3ds.com/products/catia/3dexperience-catia).

In a free run test, a set of bearings was mounted on a mild steel shaft, as illustrated in Fig. S3, and fitted in pillow blocks. One of the ends of the shafts was connected to the motor via a belt drive mechanism. The test involved measuring the vibration characteristics of the shaft supported on the fabricated DGB bearings. Each set of bearings was rotated up to 2 million cycles, and signals were captured using a 6-channel OR35-INST DAQ system integrated with ORBI gate and NV gate software via a PC system. The acceleration signals were recorded by a 1-axis ORAC-DCC-D23 accelerometer mounted on the pillow block and placed far from the motor. An ORAC-TACMM-001 optical tachometer was used to measure the speed of the shaft. Additionally, the rotor dynamics plots were recorded with the help of the eddy current probe sensors (MTN/EP080) and the DAQ system. These non-contact sensors were placed in two sets, one just beside the bearing mounted near the motor and the other away from the motor with the support of magnetic stands. Fig. 1 shows the detailed view of a testing rig used for the free run test.

### Rolling contact fatigue test

**Fig. 2:**
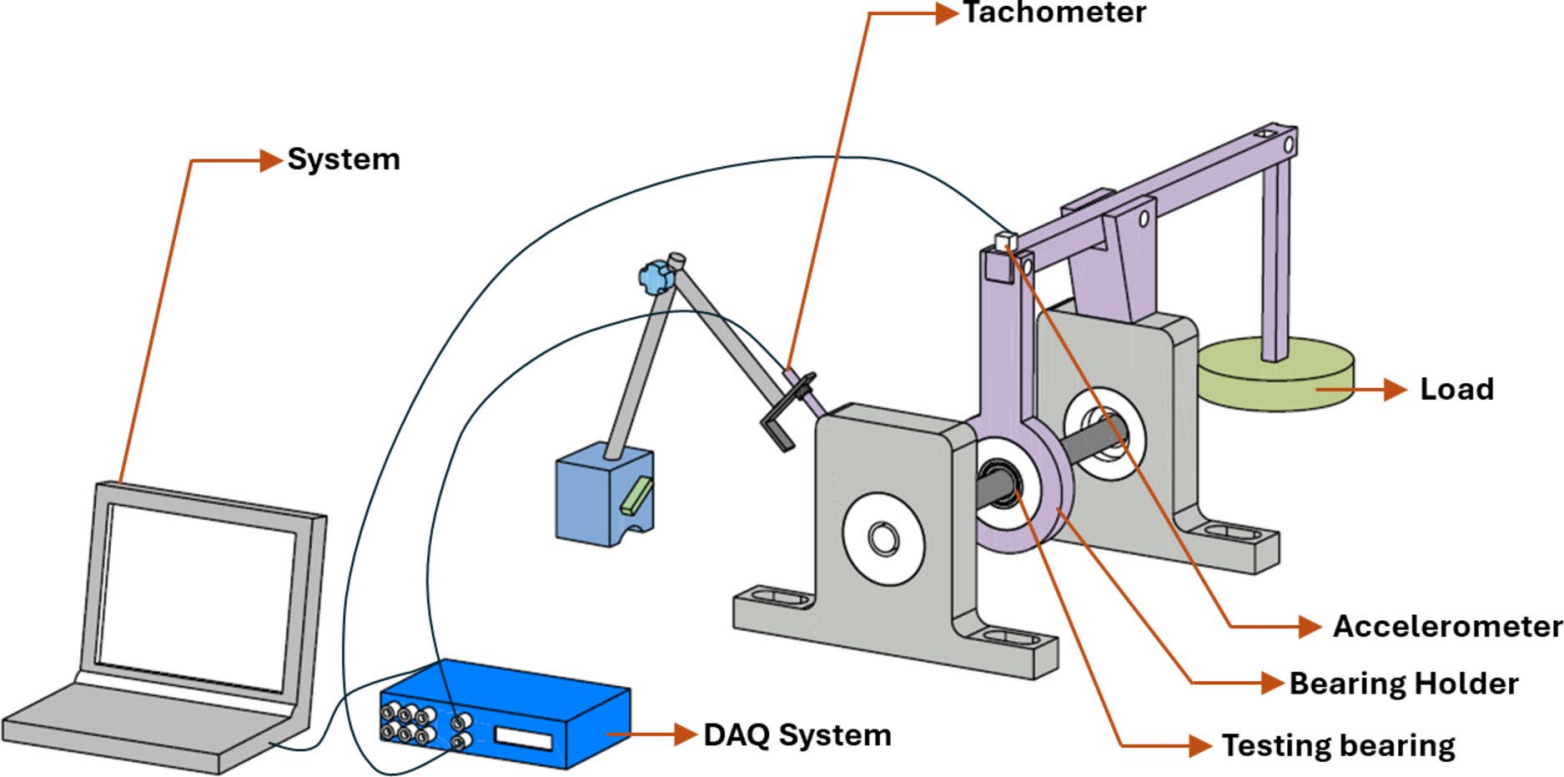
Schematic representation of RCF test rig (Generated through 3D experience https://www.3ds.com/products/catia/3dexperience-catia).

For the RCF test, an in-house developed test rig consists of two pillow blocks on which a shaft is to be mounted, a bearing holder, and metal bearings. The schematic diagram is shown in Fig. 2. A belt drive mechanism is connected to one end of the shaft to the motor. The fabricated DGB bearing was mounted on the shaft with the bearing holder. A lever was employed to apply the load on the bearing. One end of the lever was fixed on the bearing holder, and the other was subjected to the load. The accelerometer was mounted on the top of the bearing holder to record the vibrational signals. The non-contact type tachometer was mounted with the help of a magnetic stand and was used to record RPM. The bearings were rotated up to 1 million cycles at a constant speed of 800 RPM, during which they were subjected to loads ranging from 25 N to 75 N with 25 N increments. However, according to the lever rule, the actual load on the bearing ranged from 36 N to 108 N with 36 N increment. The test procedure was divided into three phases. First, the bearing was subjected to a load of 36 N up to 0.25 million cycles, and then the load was increased to 72 N for another 0.5 million cycles. Finally, for the remaining 0.25 million cycles, a load of 108 N was applied. Additionally, after completion of each phase, the bearing condition was monitored. Furthermore, after the completion of the test, the bearing race morphology was examined through FESEM.

## Results and discussion

### Ball-on-disc test

**Fig. 3:**
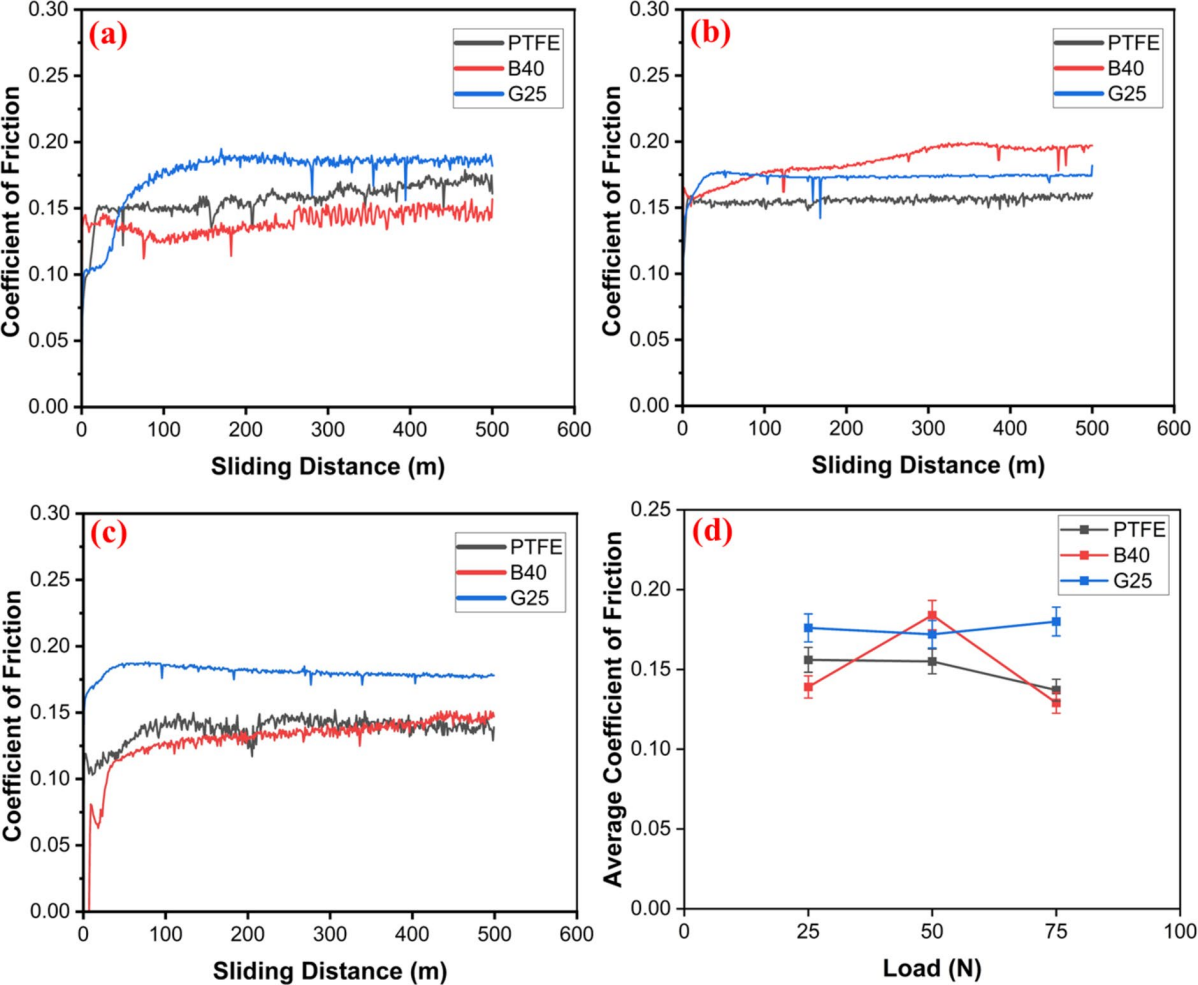
COF vs. sliding distance of the PTFE composites at different loading conditions (**a**) 25 N, (**b**) 50 N, (**c**) 75 N, (**d**) average COF.

**Fig. 4:**
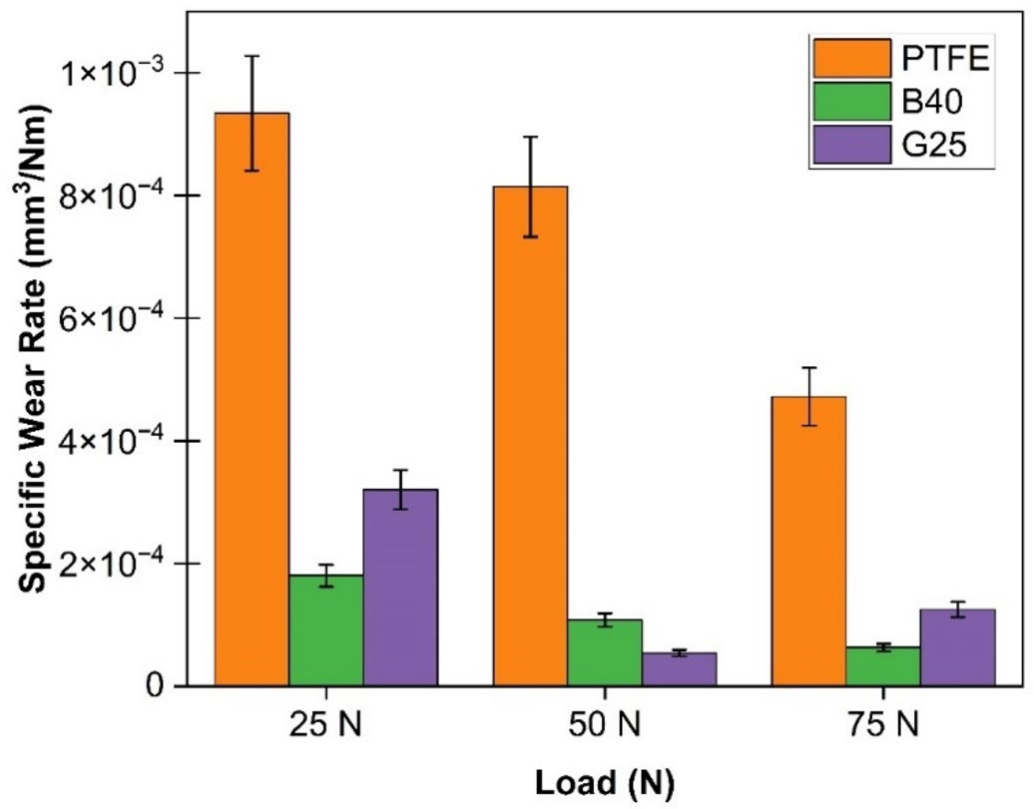
Specific wear rate of PTFE composites under different loading conditions.

Analyzing the interaction between the ball and PTFE composites is crucial to determining their suitability for ball-bearing applications. Fig. 3a–d presents the COF values of the composites under various loading conditions alongside the average COF, and Fig. 4 depicts the corresponding specific wear rate values. The test results indicate that the B40 composite demonstrates superior performance at loads of 25 N and 75 N, achieving the lowest COF and the lowest wear rate of order 10^−5^ mm^3^/Nm. In contrast, at a load of 50 N, the G25 composite shows the lowest wear rate of 5.38 × 10^−5^ mm^3^/Nm, accompanied by a COF of 0.172. Meanwhile, G25’s wear rate is slightly higher than that of B40 at 25 and 75 N. Moreover, it also reports the highest COF among all tested composites. The inherent brittleness of glass fibres contributes to their tendency for fracture, which can lead to abrasive wear, thereby increasing the COF of the composite material^41^.

Conversely, the PTFE composite demonstrates the highest wear rate among those tested, primarily due to its low hardness and Young’s modulus^42^, with COF values ranging from 0.137 to 0.156. Notably, the incorporation of bronze reinforcement has effectively reduced the wear rate by eight times. Additionally, the inclusion of glass fiber has reduced the wear rate by approximately three times. The reinforcement acts as a barrier that mitigates the fragmentation of PTFE during testing. Furthermore, it has been noticed that bronze reinforcement does not degrade the COF, although it has reduced the COF by nearly 8–10%, except at 50 N loading conditions.

**Fig. 5:**
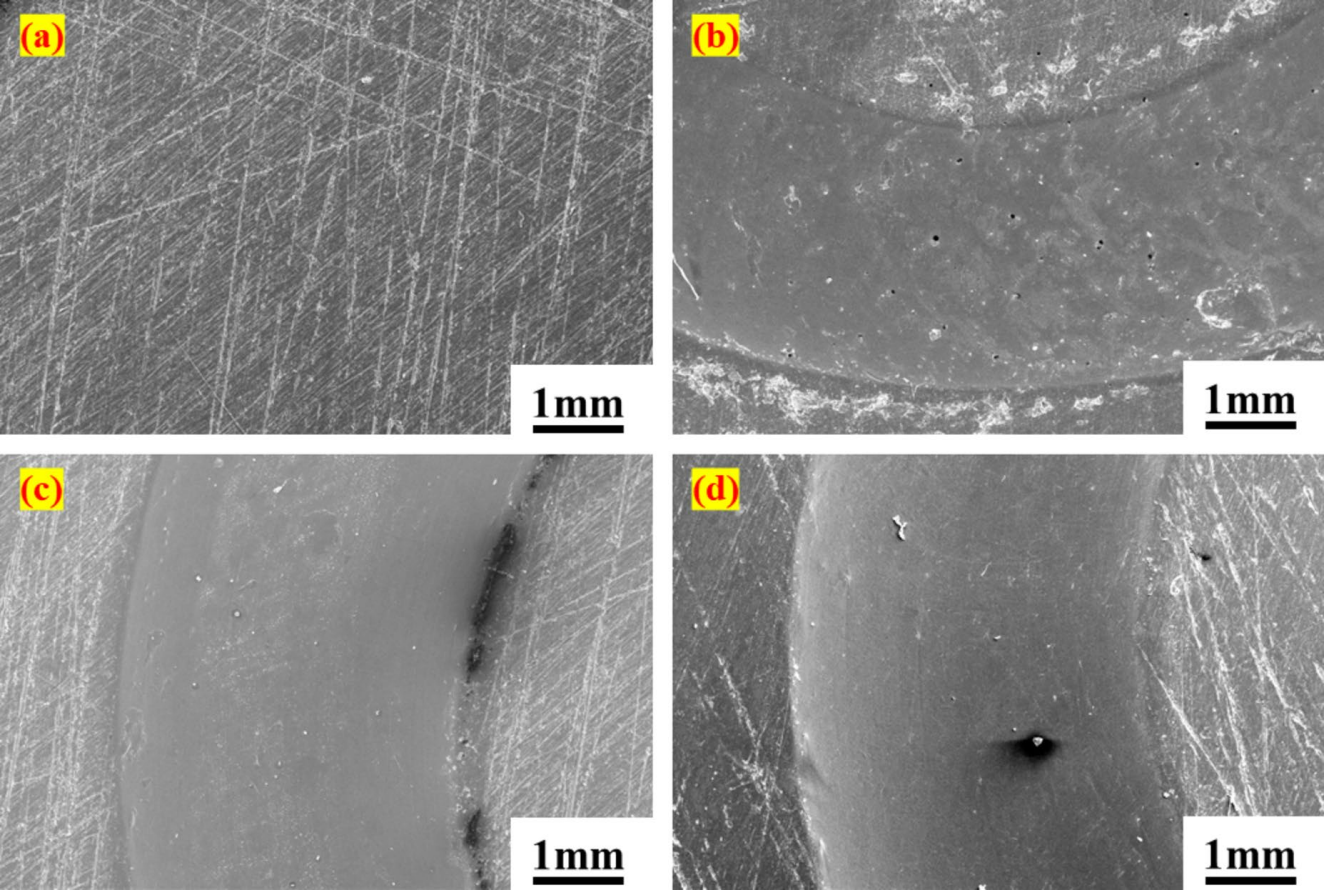
SEM images of PTFE discs wear track surface under different loading conditions (**a**) before test, (**b**) 25 N, (**c**) 50 N, (**d**) 75 N.

**Fig. 6:**
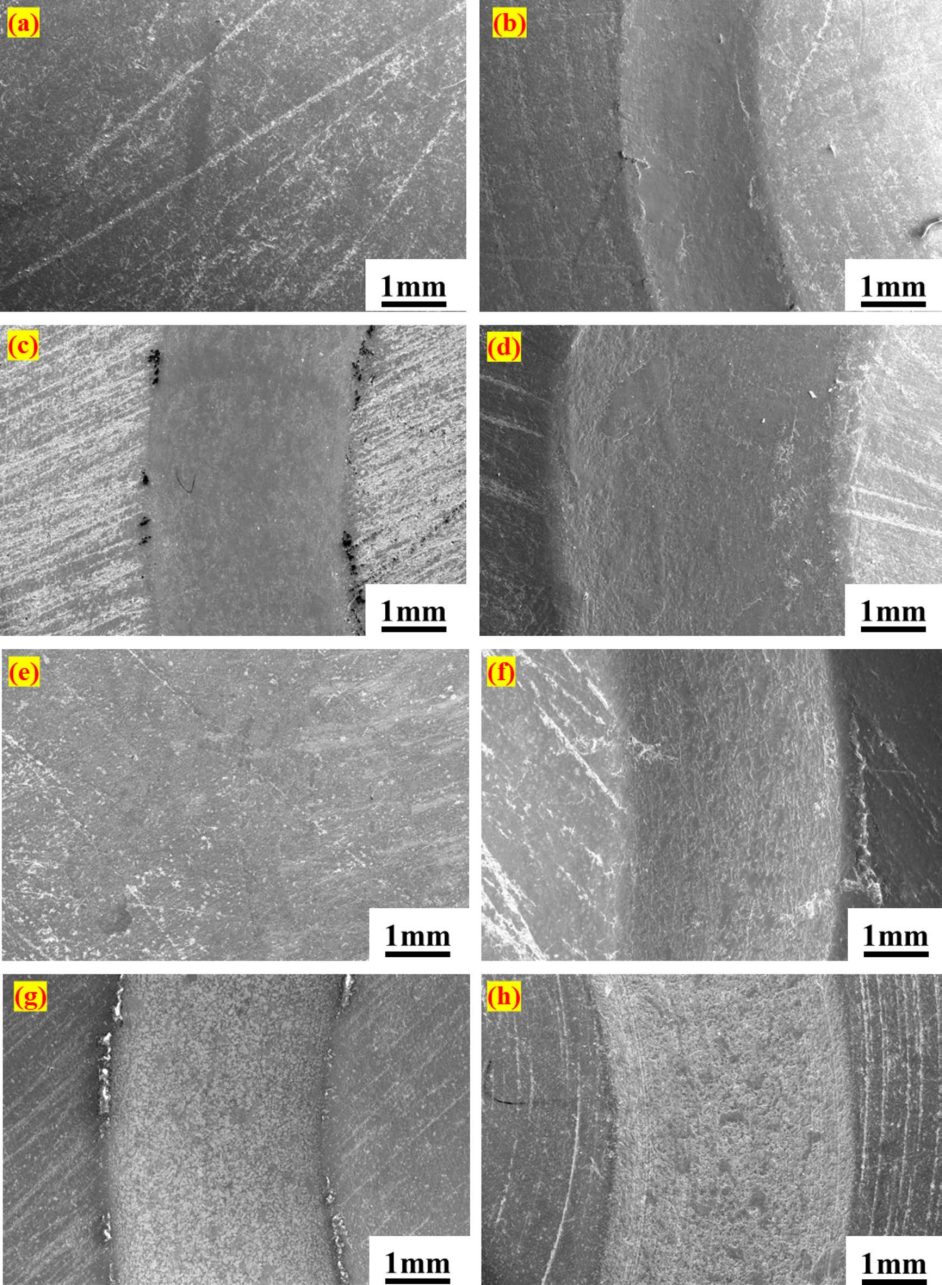
SEM images of the discs wear track surface under different loading conditions. G25 (**a**) before test, (**b**) 25 N, (**c**) 50 N, (**d**) 75 N; B40 (**e**) before test, (**f**) 25 N, (**g**) 50 N, (**h**) 75 N.

To examine the interaction between PTFE composites and Si_3_N_4_ balls, a FESEM analysis of the wear track was conducted under varying loading conditions, as presented in Figs. 5a–d and 6a–h. Fig. 5a illustrates the surface of the PTFE disc before testing, which reveals pre-existing scratches. Upon load application, the wear track exhibited a notable smoothness, leaving only a minimal wear trace due to high-stress generation at the point of interaction. As the load increased, the width of the wear track expanded from 2.527 mm at 25 N to 3.416 mm at 75 N. A similar trend was noted with the G25 disc under identical loading conditions. However, the wear track on the G25 was less smooth than that of PTFE, exhibiting visible micro-ploughing, particularly at the 75 N load, as depicted in Fig. 6b–d. This may be due to the hardness of the glass fibre. Additionally, the width of the wear track increased by up to 50% when the load was elevated from 25 N to 75 N. In the case of B40, the wear track formed on the disc under loading appeared rougher than that of the other materials, as indicated in Fig. 6f–h. The dark patches observed on the wear track of B40 are attributed to the bronze filler. Comparative analysis of the wear track widths among the different composites revealed that B40 exhibited the narrowest wear track. For instance, under 50 N loading conditions, the wear track width for B40 was measured at 2.339 mm, while PTFE registered 3.077 mm and G25 recorded 2.844 mm. Overall, the wear track results did not indicate any signs of crack formation or uneven wear. However, widening the wear track can be a major concern.

### Free run test

**Fig. 7:**
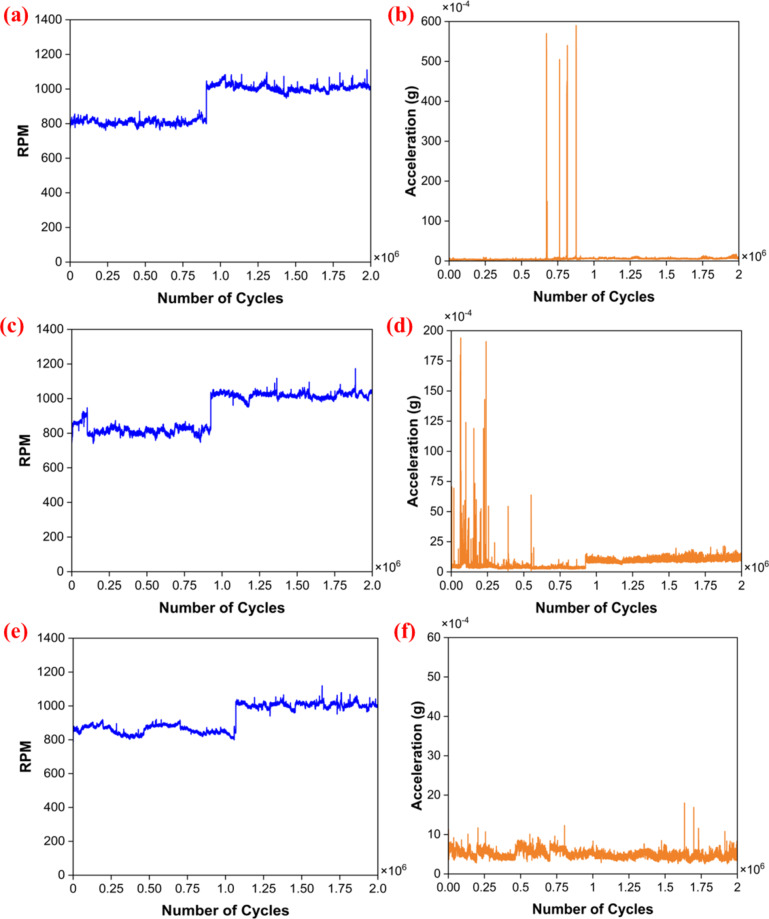
Recorded signals. PTFE (**a**) RPM, (**b**) acceleration; B40 (**c**) RPM, (**d**) acceleration; G25 (**e**) RPM, (**f**) acceleration.

In the free run test, the set of bearings was rotated for 2 million cycles under minimal load conditions, and its RPM and vibrational amplitudes were recorded, as shown in Fig. 7. During the initial 1 million cycles, each set of the bearings was rotated at 800 RPM. The PTFE bearings have shown excellent RPM stability, as shown in Fig. 7a. This indicates that the bearing ran smoothly with minimal fluctuation in RPM, and the vibration amplitude is very stable. However, sudden peaks are visible after 0.7 million cycles (Fig. 7b). The sudden spike may be due to material loss in the bearing during the run (Table 3). In the B40 bearing, there was an initial fluctuation in RPM, which later stabilized (Fig. 7c). This fluctuation led to a spike in the amplitude signal, which later stabilized, as demonstrated in Fig. 7d. The initial increase in amplitude primarily results from the interaction between the bronze particles embedded in the PTFE and the Si_3_N_4_ ball. During the early stages of testing, self-lubrication properties may not be fully effective. Direct contact between the hard materials of bronze and silicon nitride could induce some disturbances. However, as the test progresses, it is anticipated that the PTFE will gradually facilitate the transfer of a lubricating film to the counter body, enhancing the self-lubrication process. In contrast, the G25 bearing exhibited a notably low vibration amplitude, the lowest among all examined bearings (Fig. 7e). Furthermore, the RPM remained consistently stable throughout the one million cycles, as shown in Fig. 7f.

The amplitude and RPM results are favorable in the initial rotation of 1 million cycles. So, to check the reliability further, the bearing was rotated for another 1 million cycles at a higher speed of 1000 RPM. The recorded signals reveal that the vibrational amplitude exhibited by all the bearings demonstrated improved stability compared to the first million cycles. This stability is majorly due to the effective transfer of the film onto the counter body. A significant increase in RPM fluctuations was observed in all the tested bearings, which may be attributed to mass loss. This mass loss primarily results from the removal of the transfer film. Given that the bearing ball is in continuous contact with the bearing cage, the rubbing action between the ball and the cage may likely lead to removing this transfer film during rotation. Additionally, the mass loss during the free run test is summarized in Table 3, with measurements taken after every complete million cycles. The mass loss across all bearings was minimal, and no signs of bearing failure were observed. Moreover, continuous monitoring of both RPM and amplitude indicated no undesirable signal variations. Overall, after completing 2 million cycles, it can be concluded that the PTFE bearing exhibited the best RPM performance, while the G25 bearing showed superior stability in vibration amplitude.

**Table 3:** Mass loss of bearings during the free run test.

Bearing used	Position	Before the test, Wt. (g)	After 1 million cycles		After 2 million cycles	
			RPM	Wt. (g)	RPM	Wt. (g)
PTFE	Near Motor	5.7940	800	5.6681	1000	5.6164
	Far from Motor	5.7820		5.7447		5.6564
B40	Near Motor	8.000	800	7.9715	1000	7.9653
	Far from Motor	8.092		8.0522		8.0348
G25	Near Motor	6.0160	850	6.0115	1000	6.0109
	Far from Motor	6.0010		5.9987		5.9978

**Fig. 8:**
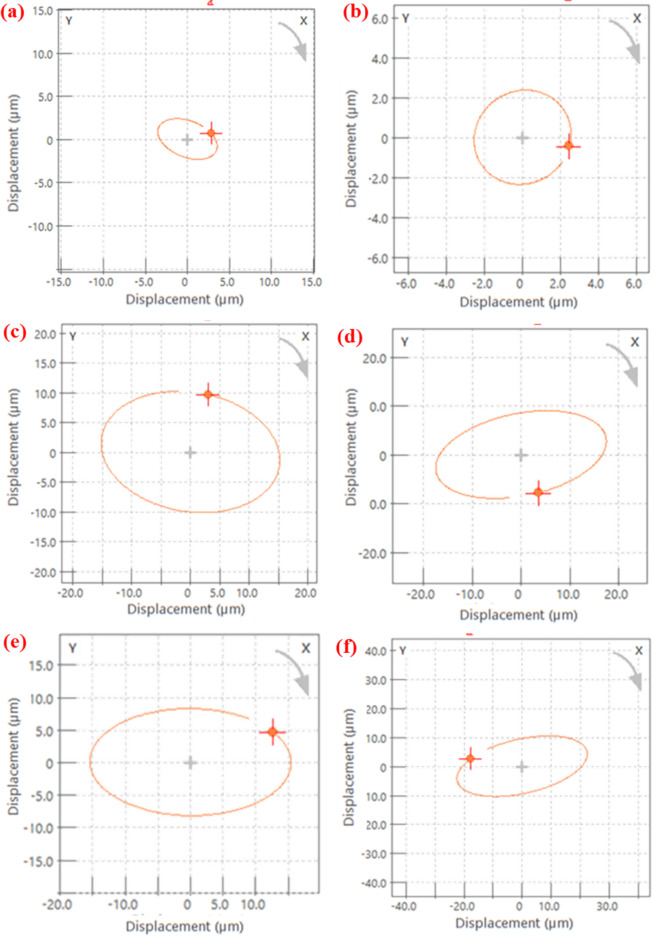
Orbit plots of a shaft supported on fabricated bearings at 720 RPM. PTFE after 1 million cycles (**a**) near the motor and (**b**) far from the motor; B40 after 0.6 million cycles (**c**) near the motor and (**d**) far from the motor; G25 after 1 million cycles (**e**) near the motor and (**f**) far from the motor.

Since the shaft dynamic behaviour is directly related to fabricated bearings performance. The test was conducted after a specified number of cycles. In the analysis, the shaft gradually rotated from 0 to 1000 RPM, and its behaviour was recorded in the form of orbit and shaft centerline plots. The dynamic characteristics were measured after 1 million cycles for the shaft supported by PTFE and G25 bearings, while plots for the B40 bearing-supported shaft were obtained after 0.6 million cycles. Orbit plots are two-dimensional representations of the shaft center during a one-cycle rotation. These plots are valuable for identifying excessive vibration within the system and misalignment in the bearings. The recorded orbit plots are presented in Fig. 8. The plots of all bearings primarily exhibit an oval shape. This phenomenon may be attributed to the lower stiffness of the bearings, and these shapes are often observed when an external force constrains the shaft’s motion.

**Fig. 9:**
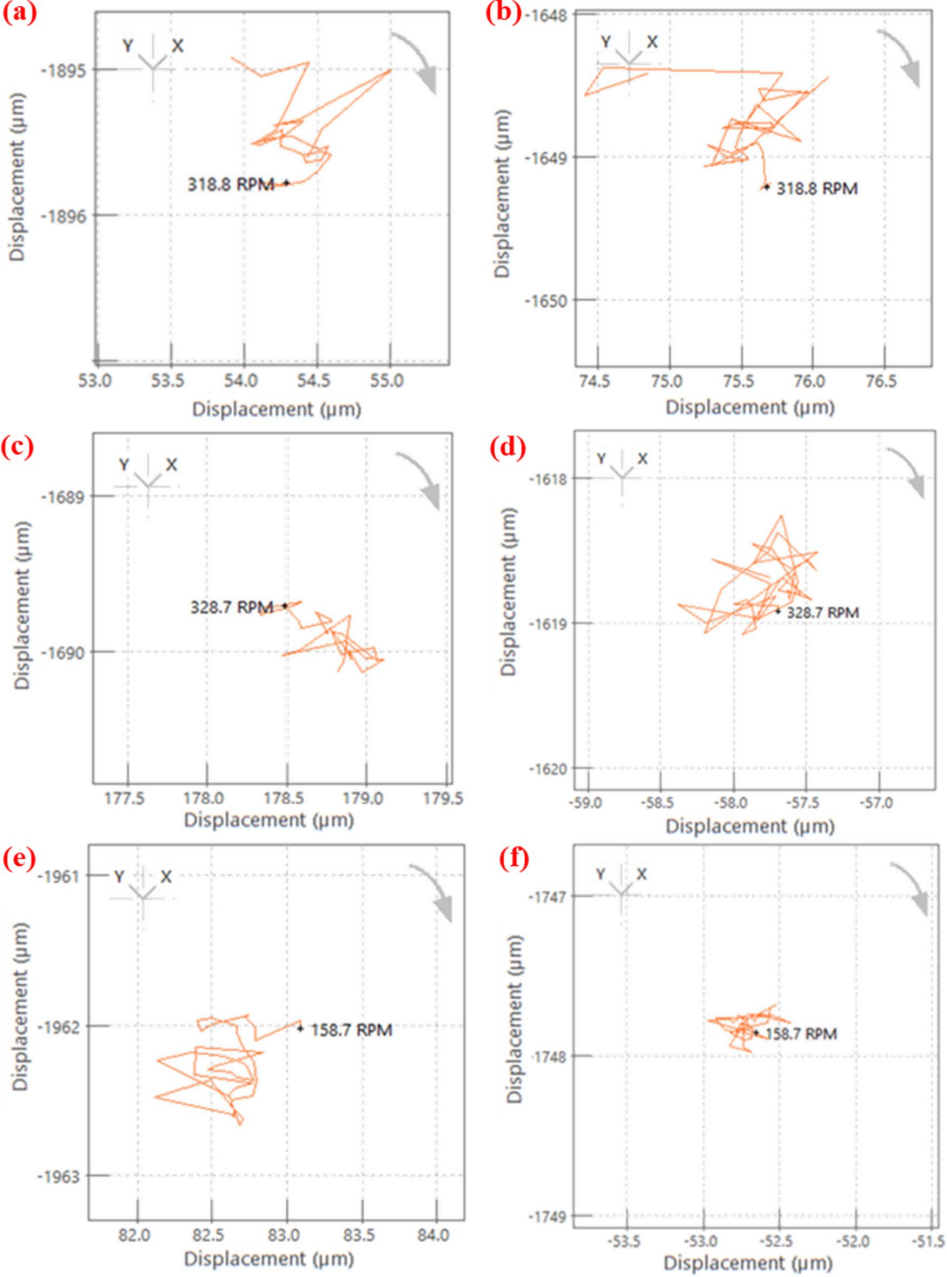
Shaft centerline plots at 720 RPM. PTFE after 1 million cycles (**a**) near the motor and (**b**) far from the motor; B40 after 0.6 million cycles (**c**) near the motor and (**d**) far from the motor; G25 after 1 million cycles (**e**) near the motor and (**f**) far from the motor.

Additionally, the shaft centerline represents the average position of the shaft center. Fig. 9 displays the shaft centerline plots for all the bearing sets, revealing that the shaft center displacement in lateral and vertical directions is less than 1 μm in all the cases. These plots signify a negligible misalignment in the shaft, and these plots can correlate with orbit plots as well. The vibration characteristics obtained during the free run test demonstrate stable performance. The orbit and centerline plots show no faults in the bearing systems with negligible misalignment.

### Rolling contact fatigue test

**Fig. 10:**
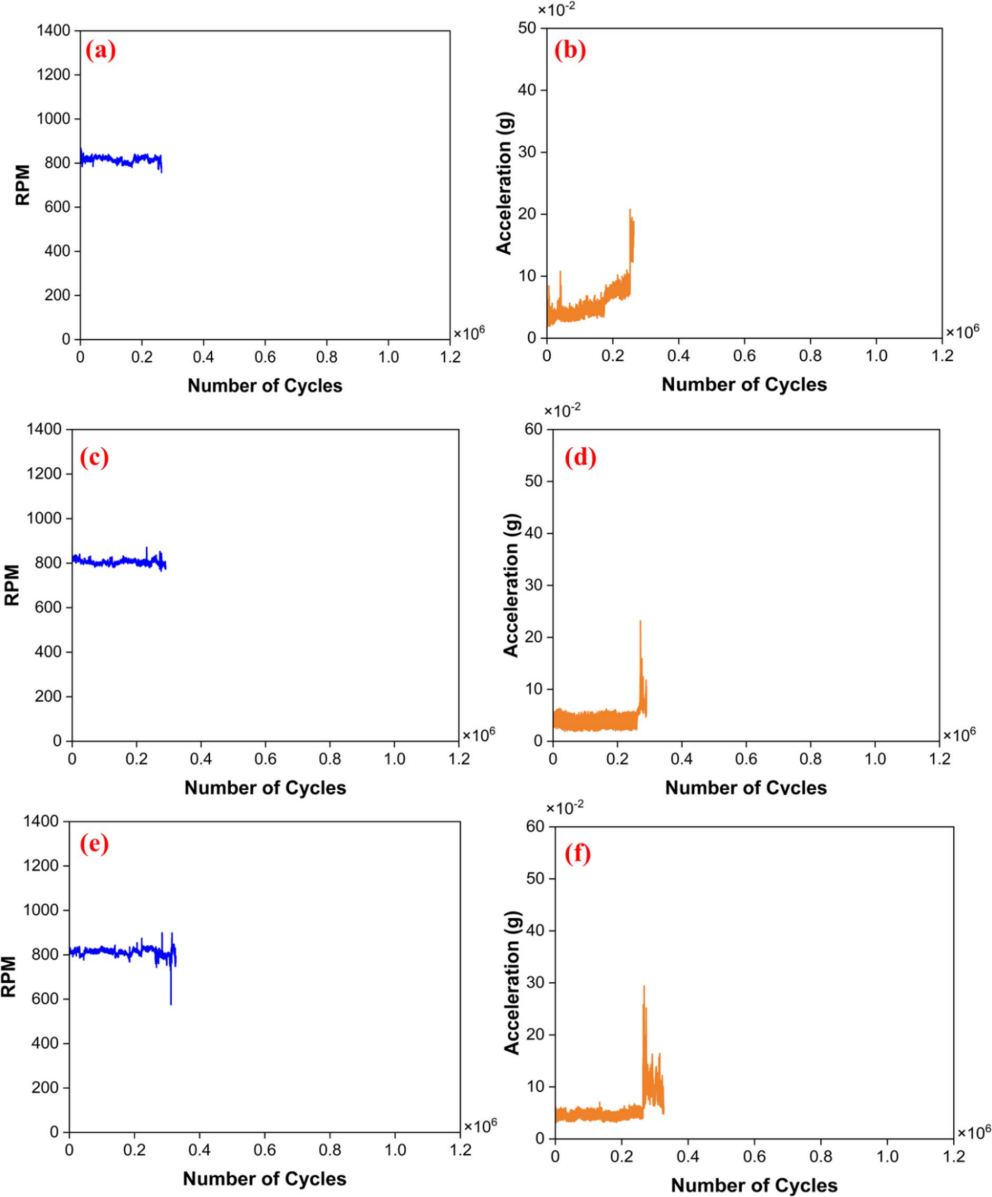
Recorded signals in RCF test; PTFE (**a**) RPM, (**b**) acceleration; G25 (**c**) RPM, (**d**) acceleration; B40 (**e**) RPM, (**f**) acceleration.

An RCF test was performed to assess the load-bearing capacity of the manufactured DGB bearings. First, the PTFE bearing was subjected to the radial load of 36 N till 0.25 million cycles. The recorded signals show no undesirable variations in the RPM, as shown in Fig. 10a. Still, a slight rise in the vibration amplitude (Fig. 10b) can be seen during the end of the 1st phase of the test. The bearing condition was assessed before the next test phase, revealing a notable gap between the bearing races and the ball. Consequently, the bearing was deemed unsuitable for further testing and failed after completing 0.25 million cycles. Then, the G25 bearing was subjected to radial load of 36 N. The initial phase of 0.25 million cycles under a load of 32 N was completed smoothly, with no discrepancies in the recorded signals. The bearing’s condition was assessed before the next phase of the test, revealing no signs of damage or misalignment. Subsequently, the bearing was subjected to a radial load of 72 N for the following 0.5 million cycles. After a certain number of cycles, a sudden rise in amplitude values was observed, accompanied by slight fluctuations in the RPM values, as illustrated in Fig. 10c, d. The test was promptly halted, and the bearing’s condition was checked. Like the PTFE, the G25 bearing failed due to the formation of a gap between the bearing race and the balls. The G25 bearing has run up to 0.26 million cycles. Lastly, the B40 bearing was tested for the initial run of 0.25 million cycles, and the bearing was able to sustain the load of 36 N with stable vibration amplitude and RPM, as seen in Fig. 10e, f. Further bearing condition was checked, and there was no sign of failure. Then, it was subjected to a 72 N load for 0.5 million cycles. Initially, the signals were stable, but later, a fluctuation in the RPM values was noted, as shown in Fig. 10e. Then, the rotation stopped, and its condition was checked. It was visible that there was a gap between the balls and the bearing races. Hence, the B40 bearing failed at 0.36 million cycles. Additionally, the mass loss observed during the test is detailed in Table 4. The mass loss in the PTFE bearing is under 1%, while the B40 and G25 bearings exhibit even lower mass losses of less than 0.2%. Notably, this mass loss is even less than the mass lost during the free run test. There was also no evidence of failure or misalignment noted during the evaluation. Therefore, while mass loss may contribute to bearing failure, it is not the primary cause.

**Fig. 11:**
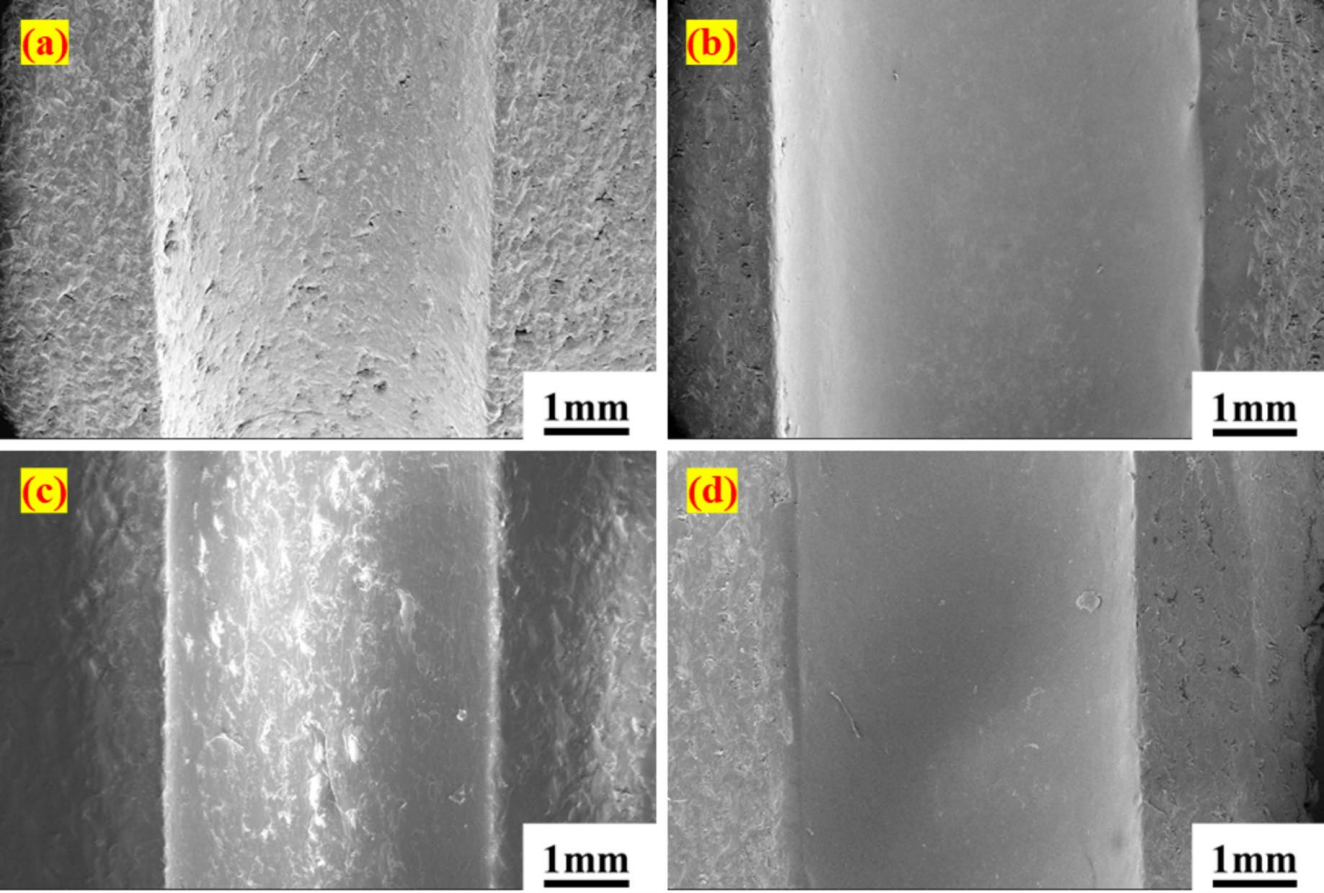
Surface morphology of PTFE bearing races, inner ring (**a**) before the test, (**b**) after the test; outer ring (**c**) before the test, (**d**) after the test.

**Fig. 12:**
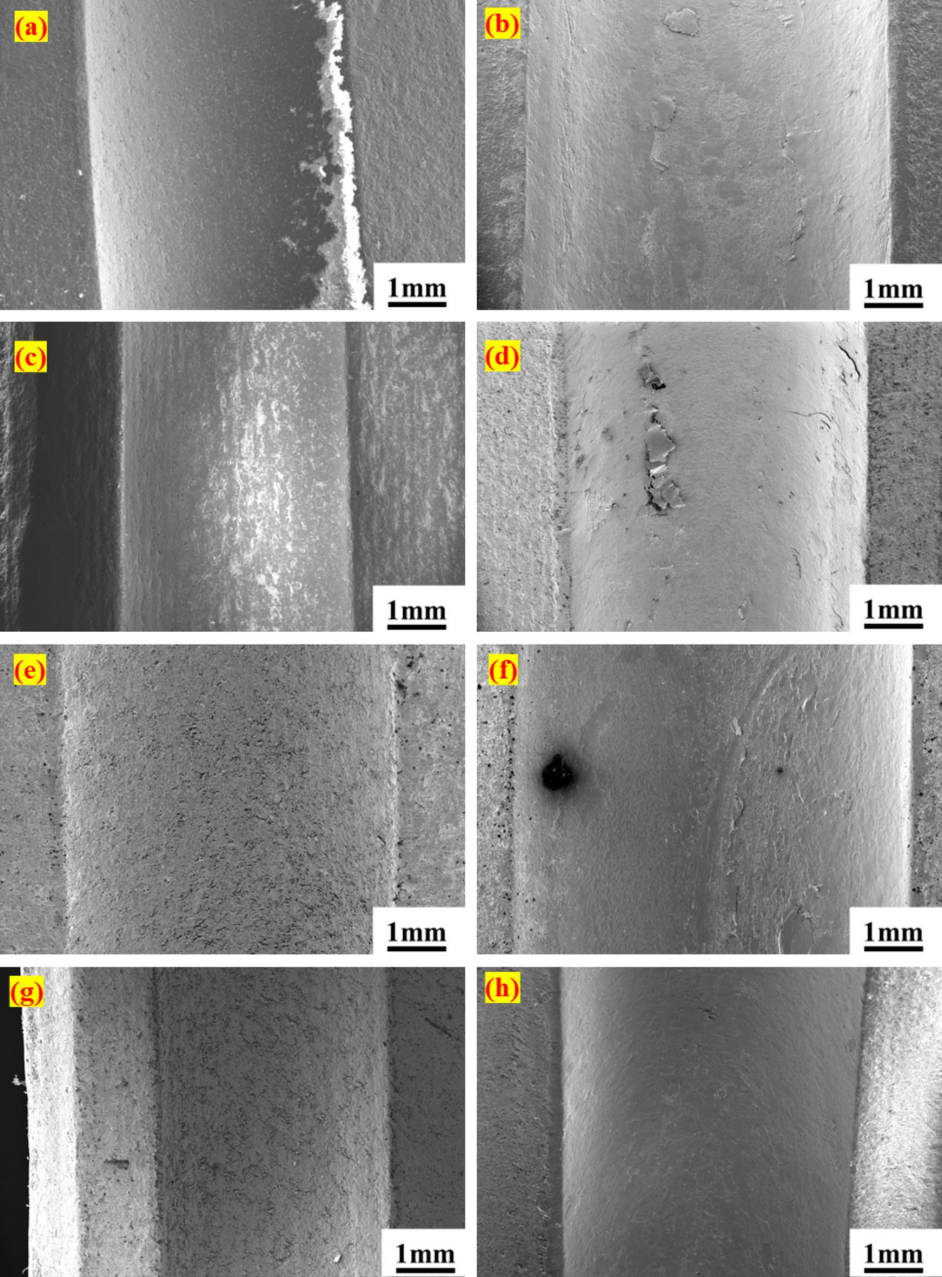
Surface morphology of bearing races. B40, inner ring (**a**) before the test, (**b**) after the test; outer ring, (**c**) before the test, (**d**) after the test. G25, inner ring (**e**) before the test, (**f**) after the test; outer ring (**g**) before the test, (**h**) after the test.

The primary cause of bearing failure, particularly in the inner ring, is the widening of the bearing race observed through FESEM. This expansion may result from the low hardness of PTFE composites and the higher hardness of silicon nitride. Additionally, failure in the bearing can be effectively detected through the analysis of bearing signals. Before testing, the width of the inner race in the PTFE bearing was measured at approximately 3.041 mm. Following the failure, this width increased to around 3.753 mm, reflecting an approximate 18% increase. Similarly, the B40 and G25 bearings experienced notable increases in the width of the inner ring race, with rises of 37% and 16%, respectively. Additionally, the outer ring race width for the B40 bearing demonstrated an approximate 16% increase, progressing from 3.081 mm to 3.60 mm. In contrast, for PTFE and G25, the outer ring race width increases were recorded at 2% and 17%, respectively. Hence, the widening of the race has created a gap between the ball and the race, which leads to failure. Furthermore, FESEM images reveal mass loss attributable to wear on the bearing races. As depicted in Fig. 11a–d, the PTFE bearings exhibited an initial rough surface attributed to machining marks during fabrication. However, post-testing observations indicate a smoothing of the surface resulting from removing these marks and the effective functioning of PTFE as a solid lubricant. However, in the case of the B40 bearings, evident wear is observed on both the inner and outer races, characterized by material ploughing due to the hardness of bronze particles, as shown in Fig. 12a–d. Similarly, for the G25 bearings, wear patterns mirrored those of the PTFE (Fig. 12e–h). Although the surface morphology of bearing races showcases no sign of cracks or breakages in the bearing, suggesting that there may not be any catastrophic failures present. Additionally, the vibration signals clearly indicated the stages of potential failure by demonstrating characteristic patterns in vibrational amplitudes.

**Table 4:** Mass loss of the bearings after the RCF test.

Bearing used	Weight before the test (g)	Weight after the test (g)	Mass loss (g)
PTFE	5.5290	5.4760	0.0530
B40	7.9860	7.9723	0.0137
G25	6.1020	6.1013	0.0007

## Conclusion

In the proposed research work, an analysis of vibration signals is carried out in the free run, and RCF tests to check the suitability of the PTFE composites for deep groove ball-bearing applications. Based on the results obtained, the following conclusions can be drawn,

- Initially, the ball-on-disc test results show that the COF between the PTFE composites and Si_3_N_4_ balls was around 0.15, which is acceptable for ball-bearing applications.
- Among the three polymer composite bearings tested, the G25 showed the best vibration stability, and PTFE showed the best RPM stability in the free-run test.
- The dynamic characteristics of the shaft mounted on the fabricated bearing sets, i.e. shaft orbit and centerline plots, show no misalignment and fault in the rotor-bearing system.
- The RCF test results reveal that PTFE, B40, and G25 bearings are suitable for ball-bearing applications in which the shaft is subjected to lower loads. Bearing race widening becomes the primary cause of failure due to the low hardness of PTFE composites in higher load conditions.
- By comparing all the results obtained, it can be concluded that reinforcement can improve the bearing’s performance of PTFE. However, there is a scope for improvement in the PTFE hardness.
- There was no catastrophic failure in the bearings, which is a good sign for any system. The instability caused by these bearings was easily detectable through the vibration amplitude signals.
- The reinforcement in hybrid forms shall be considered. The synergistic effects of combining multiple fillers have been shown to significantly enhance the performance of PTFE, leading to an overall improvement in its properties.
- Hybrid-based PTFE composites can be selected for better performance in ball-bearing applications.

## Electronic supplementary material

Below is the link to the electronic supplementary material.

Supplementary Material 1

## Data Availability

Data to support the findings of this study are available with the corresponding author and made available upon reasonable request.
