# Supplementary material for: Assessment of polytetrafluoroethylene composites for deep groove ball bearing applications through free run and rolling contact fatigue tests
Source: Sci Rep. 2025 Mar 24;15:10088. doi: 10.1038/s41598-025-94547-4 (PMC11933675; doi:10.1038/s41598-025-94547-4)
Supplement: Supplementary file 1 — Supplementary Material 1 [file 41598_2025_94547_MOESM1_ESM.docx]

**Supplementary Material**


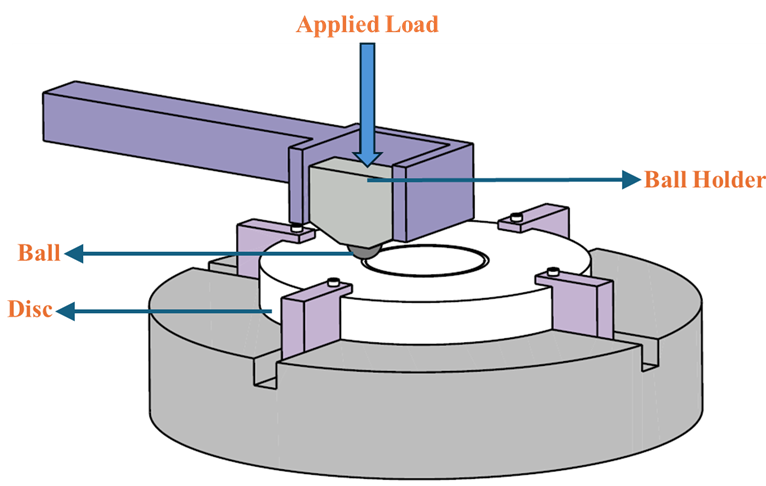


**Figure S1:** Schematic diagram of Ducom TR-20 NEO series friction and wear monitor for ball-on-disc test (generated through CATIA 3Dexperience https://www.3ds.com/products/catia/3dexperience-catia).


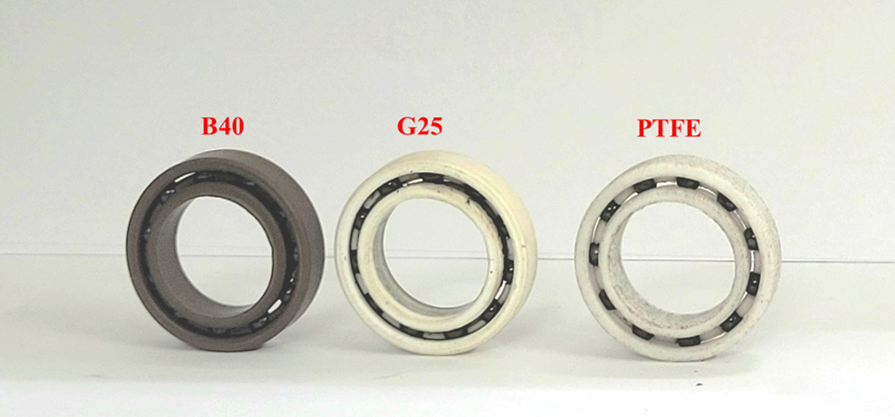


**Figure S2:** PTFE, B40 and G25 deep groove ball bearings.


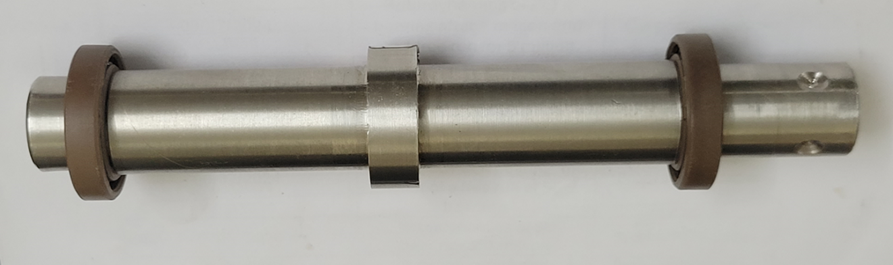


**Figure S3:** B40 deep groove ball bearings mounted on the shaft.
